# Supplementary figures and images for: Conserved shifts in sperm small non-coding RNA profiles during mouse and human aging
Source: EMBO J. 2026 Jan 20;45(4):1362–80. doi: 10.1038/s44318-025-00687-8 (PMC12909834; doi:10.1038/s44318-025-00687-8)

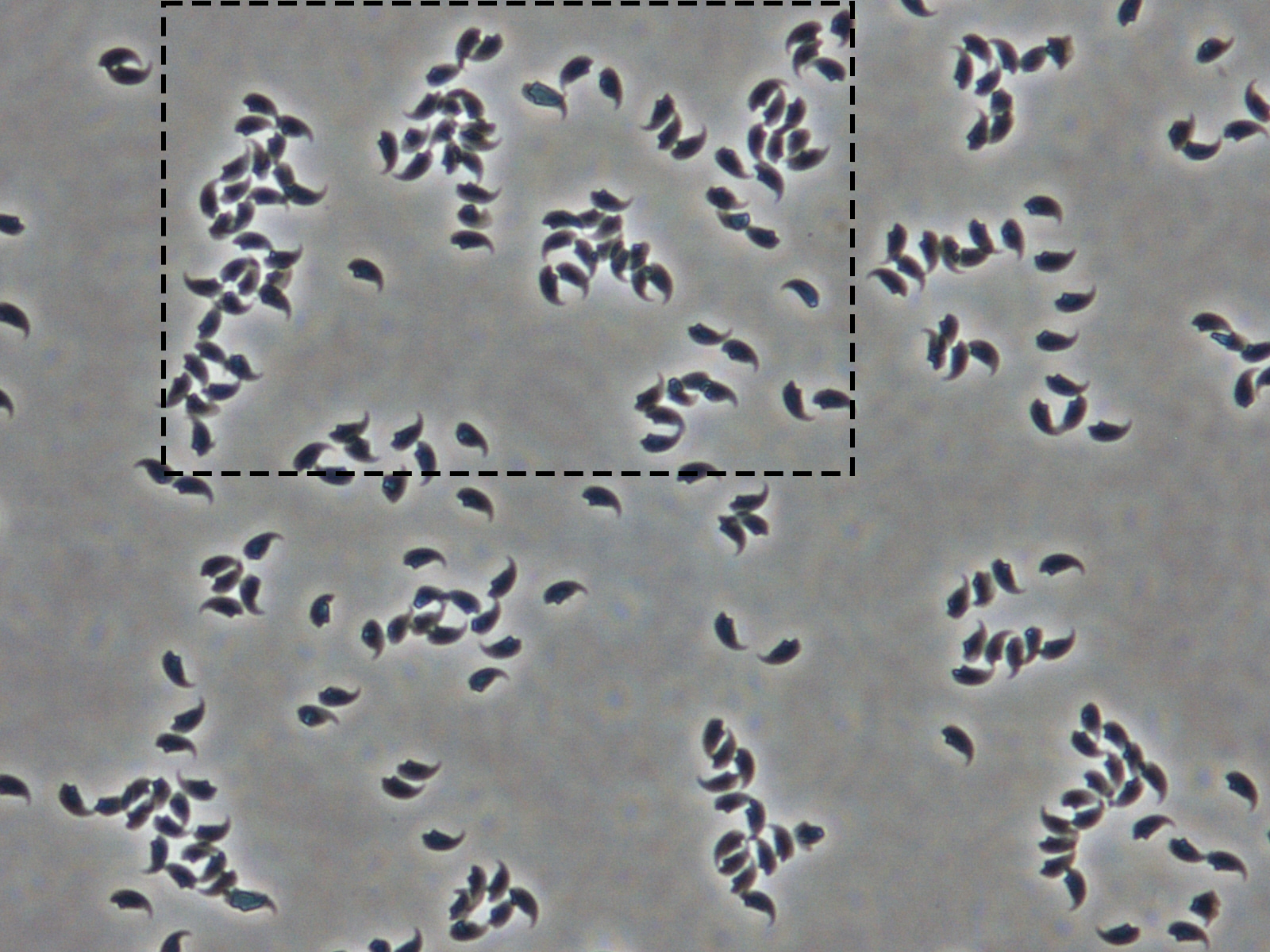

Supplement: Supplementary file 14 — Source data Fig. 1 [file 44318_2025_687_MOESM14_ESM.zip › 1F/Fig.1F_purifed sperm heads x40.tif]
