# Supplementary material for: Conserved shifts in sperm small non-coding RNA profiles during mouse and human aging
Source: EMBO J. 2026 Jan 20;45(4):1362–80. doi: 10.1038/s44318-025-00687-8 (PMC12909834; doi:10.1038/s44318-025-00687-8)
Supplement: Supplementary file 18 — Expanded View Figures [file 44318_2025_687_MOESM18_ESM.pdf]

Expanded View Figures

Figure EV1. miRNA "aging cliff".

miRNA profiles detected by PANDORA-seq show an aging cliff in intact sperm (**A–D**) and sperm heads (**E–H**) during mouse aging but is less prominent than the tsRNA/rsRNA profiles. (**A**) Illustrative figure showing the intact sperm collection at five time points (10-, 30-, 50-, 70-, and 90-week) during mouse aging. (**B**, **C**) Principal coordinate analysis (PCoA) of mouse sperm miRNA profiles showing that (**B**) PANDORA-seq, but not (**C**) traditional sncRNA-seq, identified an "aging cliff" during the 50–70-week transition in mouse sperm. Axis 1: the first principal coordinate; Axis 2: the second principal coordinate. (**D**) The ratio of between-group variance to within-group variance (*F*-statistic) is significantly higher in tsRNA/rsRNA group than that of the miRNA group in PANDORA-seq data, supporting that tsRNA/rsRNA profile shows a better classification power than miRNA profile between the demarcated stages (early 10-/30-/50-week vs. later 70-/90-week). The *P* value was computed by Wilcoxon test. (**E–H**) similar analyses on purified sperm heads as those of (**A–D**). For boxplot in (**D**, **H**), the bold horizontal line indicates the median of the data. The lower and upper box boundaries of the box indicate the 25th percentile (Q1) and 75th percentile (Q3) of the data, respectively. Accordingly, the interquartile range (IQR) is Q3-Q1. The lower and upper whiskers indicates the most extreme data points that fall within Q1-1.5 × IQR and Q3 + 1.5 × IQR, respectively. For (**D**): $n = 1005$ for miRNA and $n = 81$ for tsRNA/rsRNA; For (**H**): $n = 950$ for miRNA and $n = 81$ for tsRNA/rsRNA

 © The Author(s)

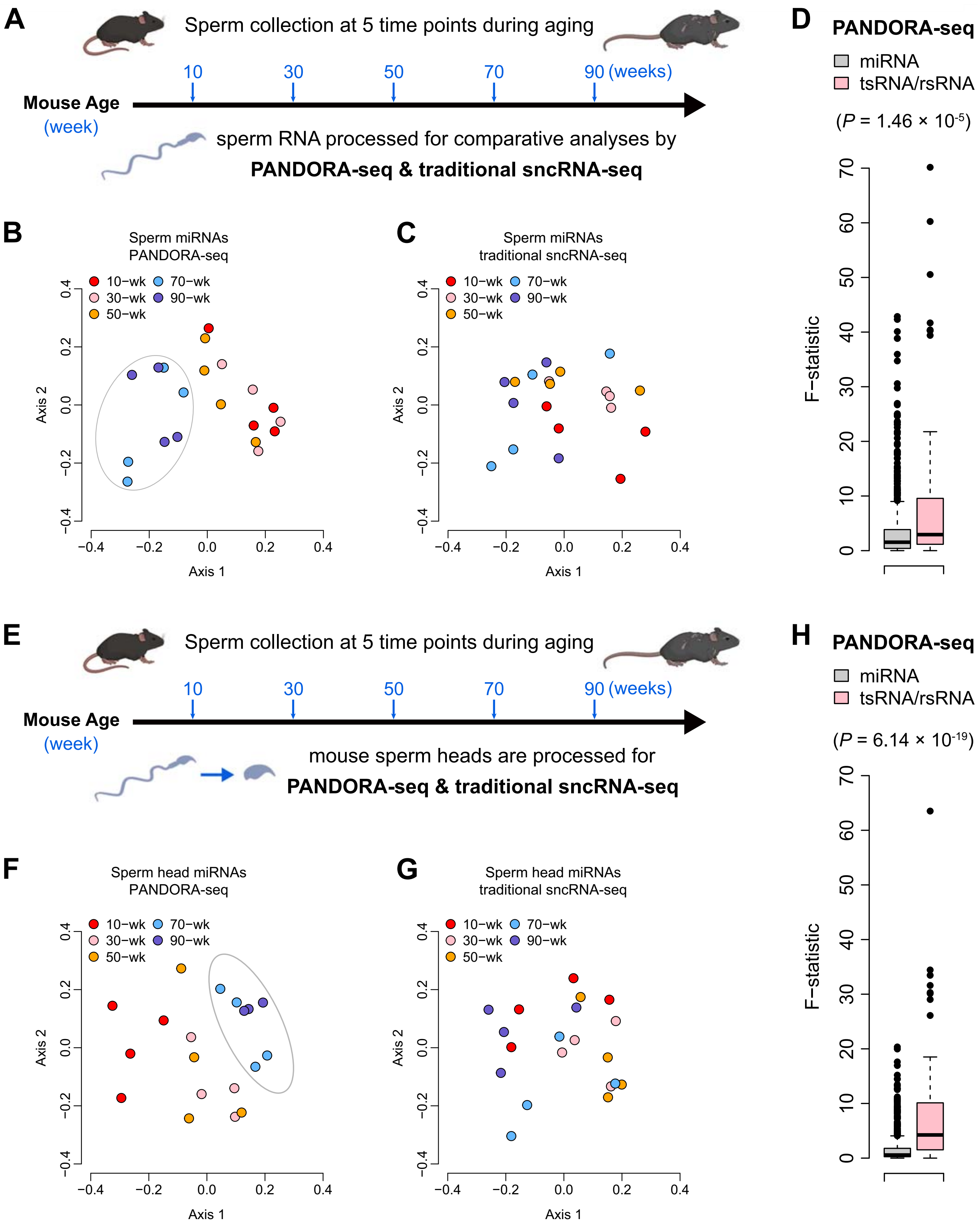
A
Sperm collection at 5 time points during aging
10
30
50
70
90 (weeks)
Mouse Age
(week)
sperm RNA processed for comparative analyses by
PANDORA-seq & traditional sncRNA-seq
B
Sperm miRNAs
PANDORA-seq
10−wk
70−wk
30−wk
90−wk
50−wk
Axis 2
Axis 1
C
Sperm miRNAs
traditional sncRNA-seq
D
PANDORA-seq
miRNA
tsRNA/rsRNA
(P = 1.46 × 10-5)
F−statistic
E
Sperm collection at 5 time points during aging
mouse sperm heads are processed for
PANDORA-seq & traditional sncRNA-seq
F
Sperm head miRNAs
PANDORA-seq
G
Sperm head miRNAs
traditional sncRNA-seq
H
PANDORA-seq
miRNA
tsRNA/rsRNA
(P = 6.14 × 10-19)
F−statistic

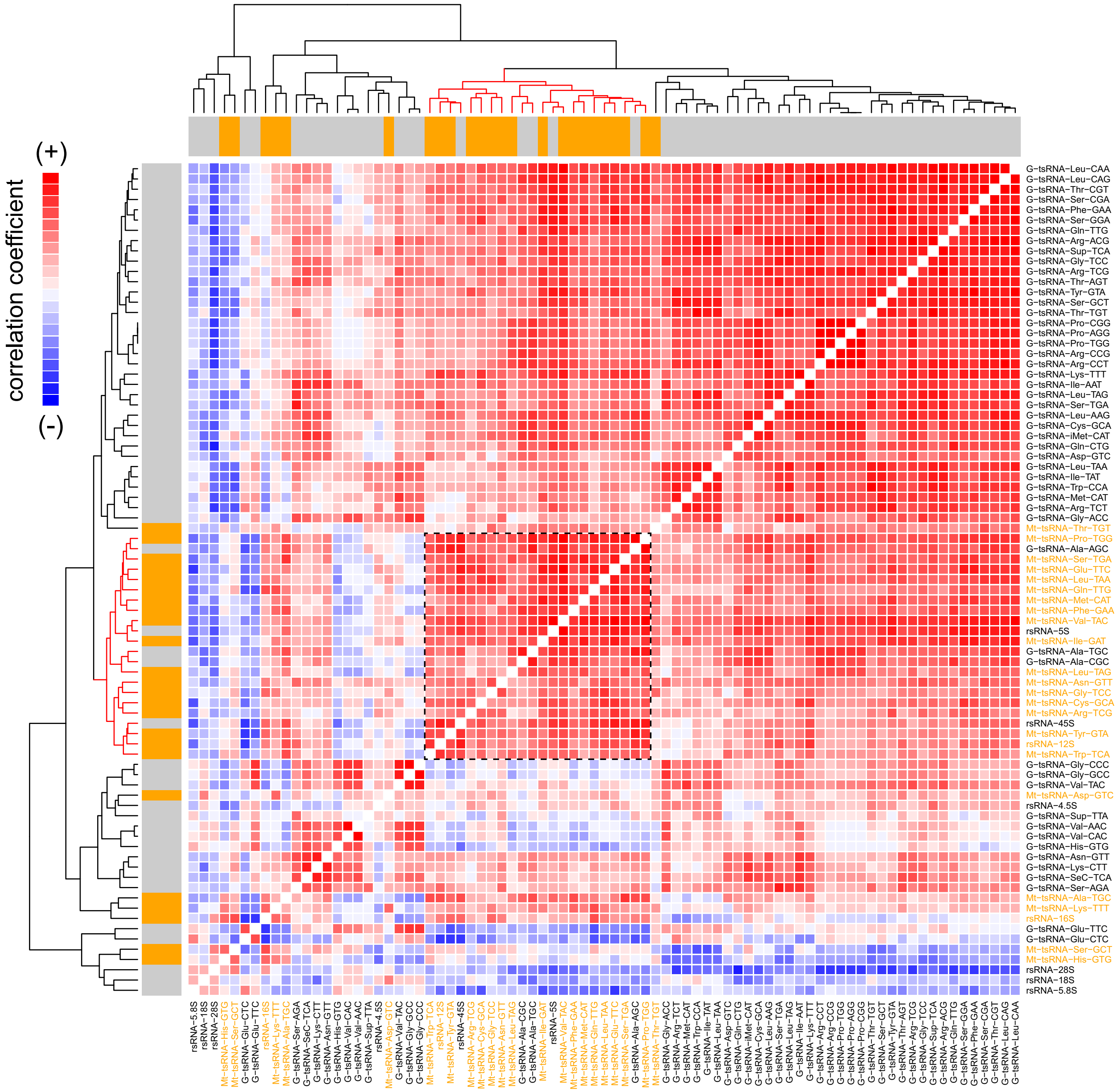

Figure EV2. Co-expression pattern of all the genomic tsRNAs/rsRNAs and mitochondrial tsRNAs/rsRNAs in the mouse de-membranated sperm heads.

The colors in the heatmap represent the intensity of co-expression (i.e., Spearman's rank correlation coefficient) between the sncRNAs: red indicates positive co-expression, while blue indicates negative co-expression. This co-expression pattern is a magnified version of Fig. 1G, showing the identity of each tsRNA/rsRNA category on the heatmap. The enriched mt-tsRNA/rsRNA cluster (the dashed area in the middle) suggests that these mitochondrial sncRNAs are transcribed or regulated in a coordinated manner, which may reflect that, in the mature sperm, the genomic transcription is silent while the mitochondrial DNA transcription remains active. G-tsRNA genomic tsRNA, Mt-tsRNA mitochondrial tsRNA.

 © The Author(s)

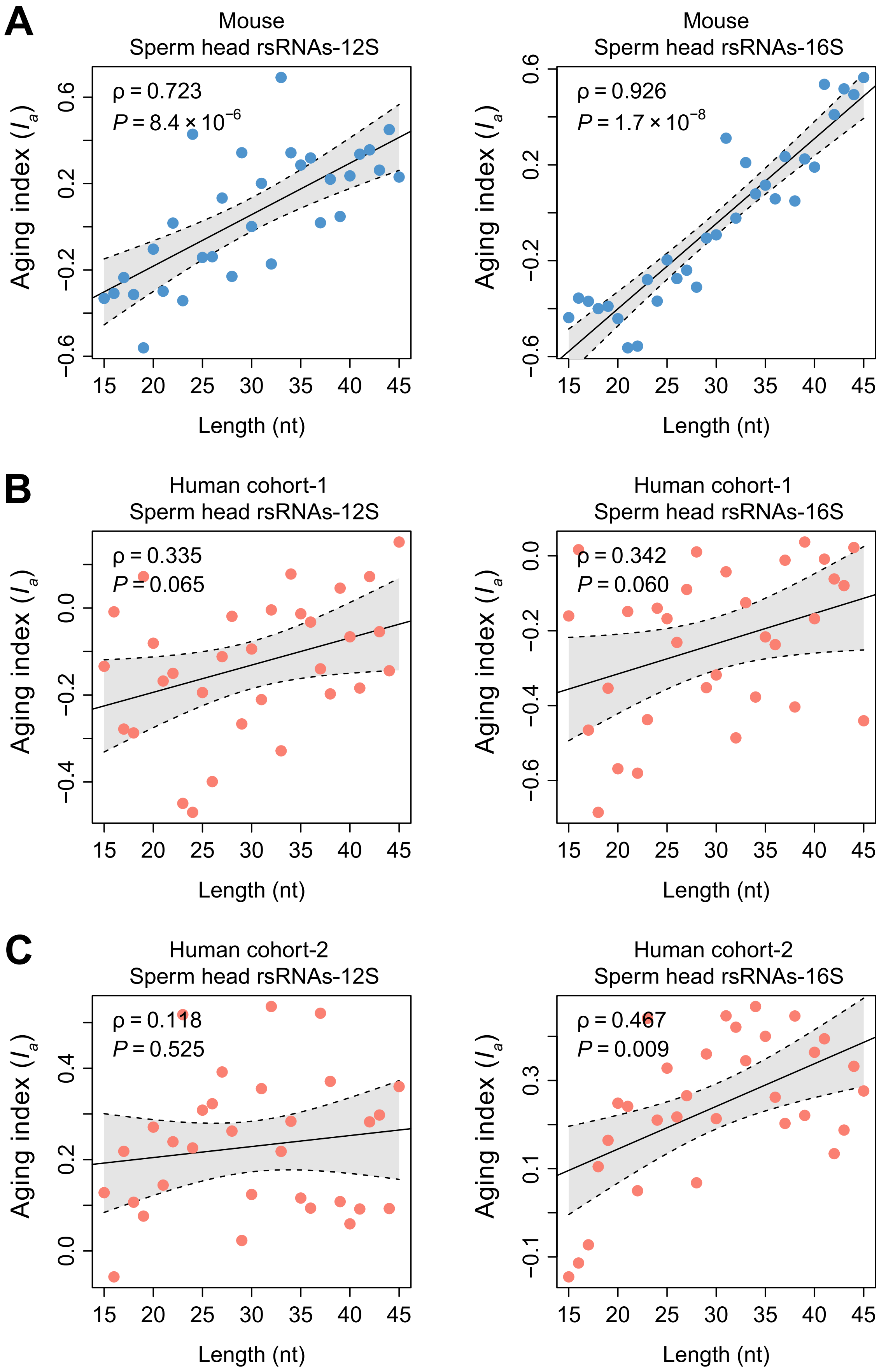
A
Mouse
Sperm head rsRNAs-12S
ρ = 0.723
P = 8.4 × 10−6
Mouse
Sperm head rsRNAs-16S
ρ = 0.926
P = 1.7 × 10−8
B
Human cohort-1
Sperm head rsRNAs-12S
ρ = 0.335
P = 0.065
Human cohort-1
Sperm head rsRNAs-16S
ρ = 0.342
P = 0.060
C
Human cohort-2
Sperm head rsRNAs-12S
ρ = 0.118
P = 0.525
Human cohort-2
Sperm head rsRNAs-16S
ρ = 0.467
P = 0.009
Aging index (Ia)
Length (nt)

◀ **Figure EV3. Age-related length shift of mitochondrial rsRNAs (12S, 16S) in mouse and human sperm heads.**

RNA sample was extracted from de-membranated sperm heads from (**A**) mouse during 10–90 weeks as that of Fig. 2A, (**B**) human cohort-1, and (**C**) human cohort-2 as that of Fig. 3A, B, followed by PANDORA-seq. We calculated the association of expression (RPM) with age (Spearman correlation), which we termed as aging index (I_a). Each dot represents the value of I_a for the corresponding length. The scatter plots demonstrate the relationship between I_a and RNA length, which was measured by Spearman's rank correlation coefficient (ρ) and the corresponding P value. The solid lines depict linear regression fits.

 © The Author(s)

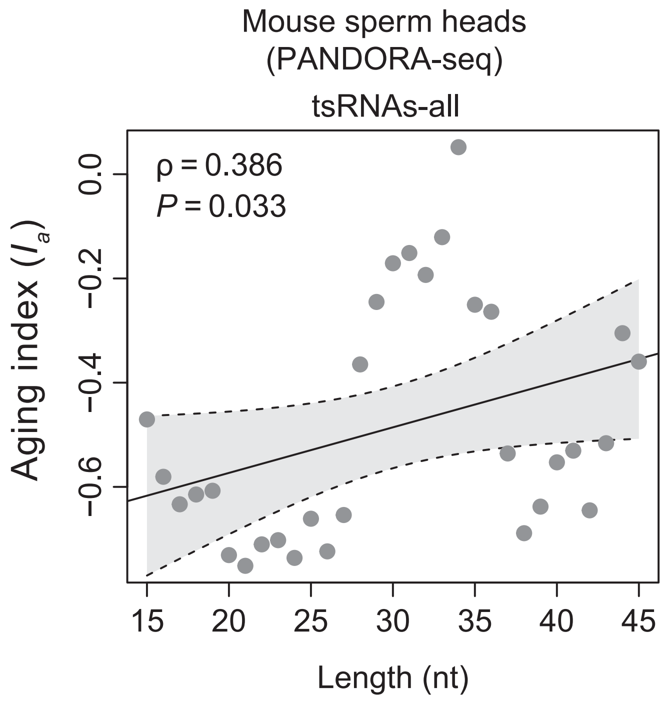

Figure EV4. Less prominent age-related length shift of tsRNAs in mouse sperm heads.

Age-related length shift analyses of tsRNAs are similarly performed as that of rsRNAs in Fig. 2A, where sncRNAs were extracted from de-membranated sperm heads followed by PANDORA-seq. We calculated the association of expression (RPM) with age (Spearman's rank correlation), which we termed as aging index (I_a). Each dot represents the value of I_a for the corresponding length. The scatter plots demonstrate the relationship between I_a and RNA length, which was measured by Spearman's rank correlation coefficient (ρ) and the corresponding *P*-value. The solid lines depict linear regression fits. The figure shows that the age-related length shift in tsRNAs is less prominent compared to that of rsRNAs in sperm heads.

© The Author(s)
